# Trends in Respiratory Virus Infections During the COVID-19 Pandemic in Singapore, 2020

**DOI:** 10.1001/jamanetworkopen.2021.15973

**Published:** 2021-06-28

**Authors:** Wei Yee Wan, Koh Cheng Thoon, Liat Hui Loo, Kian Sing Chan, Lynette L. E. Oon, Adaikalavan Ramasamy, Matthias Maiwald

**Affiliations:** 1 Department of Microbiology, Singapore General Hospital, Singapore; 2 Infectious Disease Service, Department of Paediatric Medicine, KK Women’s and Children’s Hospital, Singapore; 3 Lee Kong Chian School of Medicine, Nanyang Technological University, Singapore; 4 Duke–National University of Singapore Graduate Medical School, Singapore; 5 Yong Loo Lin School of Medicine, National University of Singapore, Singapore; 6 Department of Pathology and Laboratory Medicine, KK Women’s and Children’s Hospital, Singapore; 7 Department of Molecular Pathology, Singapore General Hospital, Singapore; 8 Genome Institute of Singapore, Agency for Science, Technology, and Research (A*STAR), Singapore

## Abstract

This cross-sectional study assessed the changes in respiratory virus prevalence in 2020 vs 2019 associated with the COVID-19 pandemic.

## Introduction

The COVID-19 pandemic brought unprecedented challenges to the world. Many jurisdictions implemented control measures, such as border closures, lockdowns, school and business closures, travel restrictions, mask wearing, and social distancing. This was associated with changes in the prevalence of other respiratory viruses, predominantly influenza viruses^1,2^ but others as well.^3^

Singapore represents a unique setting that is credited with having a successful COVID-19 response. It went through different response phases, from prelockdown (pandemic level 3) to a full lockdown (known as circuit breaker), followed by a phased reopening, during which schools and businesses reopened but social distancing measures and universal mask wearing remained in place (eTable in the Supplement). Our aim in this study was to assess the associated changes in respiratory virus prevalence in 2020 compared with the prepandemic year 2019.

## Methods

In this cross-sectional study, data from respiratory virus multiplex polymerase chain reaction tests from 3 major public hospitals in Singapore for 2019 and 2020 were compiled (eMethods in the Supplement). This included hospital A, a women’s and children’s hospital (800 beds); hospital B, offering pediatric and adult services (1200 beds); and hospital C, with adult services (1700 beds). Weekly numbers of tests and positive results for each virus were collated. Institutional review board application and informed consent were not required under SingHealth institutional rules. We followed the Strengthening the Reporting of Observational Studies in Epidemiology (STROBE) reporting guideline. A 1-tailed *t *test (with significance set at *P* = .05) was used to compare means. Data were compiled using Excel 2013 (Microsoft) and analyzed using R version 3.6.2 (R Project for Statistical Computing).

## Results

**Figure 1.**
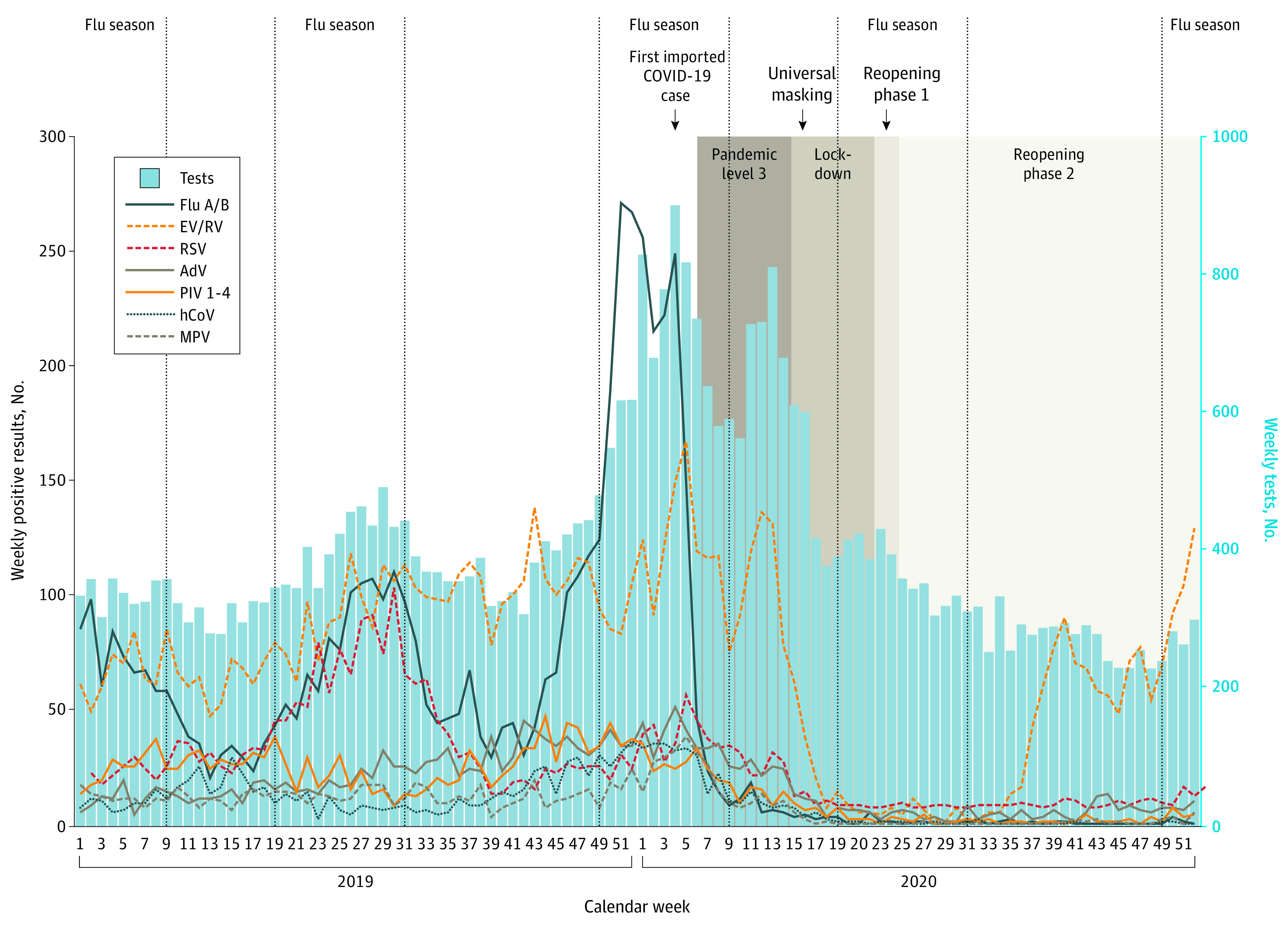
Numbers of Respiratory Virus Tests and Positive Tests for Different Viruses in 2019 and 2020 Blue bars indicate weekly numbers of multiplex tests performed, and colored lines indicate positive tests for different viruses per calendar week in 2019 and 2020. Influenza (flu) seasons are denoted by vertical lines according to Singapore Health Hub.^4^ AdV indicates adenovirus; EV/RV, enterovirus/rhinovirus (combined test); hCoV, common cold coronaviruses HKU1, NL63, 229E, and OC43; MPV, human metapneumovirus; PIV 1-4, parainfluenza viruses 1-4; and RSV, respiratory syncytial virus.

The full data set comprised 42 558 test results, 19 898 (46.8%) from 2019 and 22 660 (53.2%) from 2020. During 2019, influenza A/B, enterovirus/rhinovirus, and respiratory syncytial virus (RSV) were most commonly detected (Figure 1).^4^ A peak of influenza activity occurred during December 2019 to January 2020 (prepeak, week 33 to 45, 2019: mean [SD], 12.9 [2.7] cases per 100 samples; peak, week 46, 2019, to week 5, 2020: mean [SD], 29.8 [7.5] cases per 100 samples; *P* < .001). Enterovirus/rhinovirus activity was high starting in the second half of 2019 (week 1 to 25, 2019: mean [SD], 20.4 [2.6] cases per 100 samples; week 26 to 52, 2019: mean [SD], 25.2 [5.2] cases per 100 samples; *P* < .001). Adenovirus activity increased starting in approximately September 2019 (week 1 to 38, 2019: mean [SD], 4.6 [1.6] cases per 100 samples; week 39 to 52, 2019: mean [SD], 8.5 [2.5] cases per 100 samples; *P* < .001).

**Figure 2.**
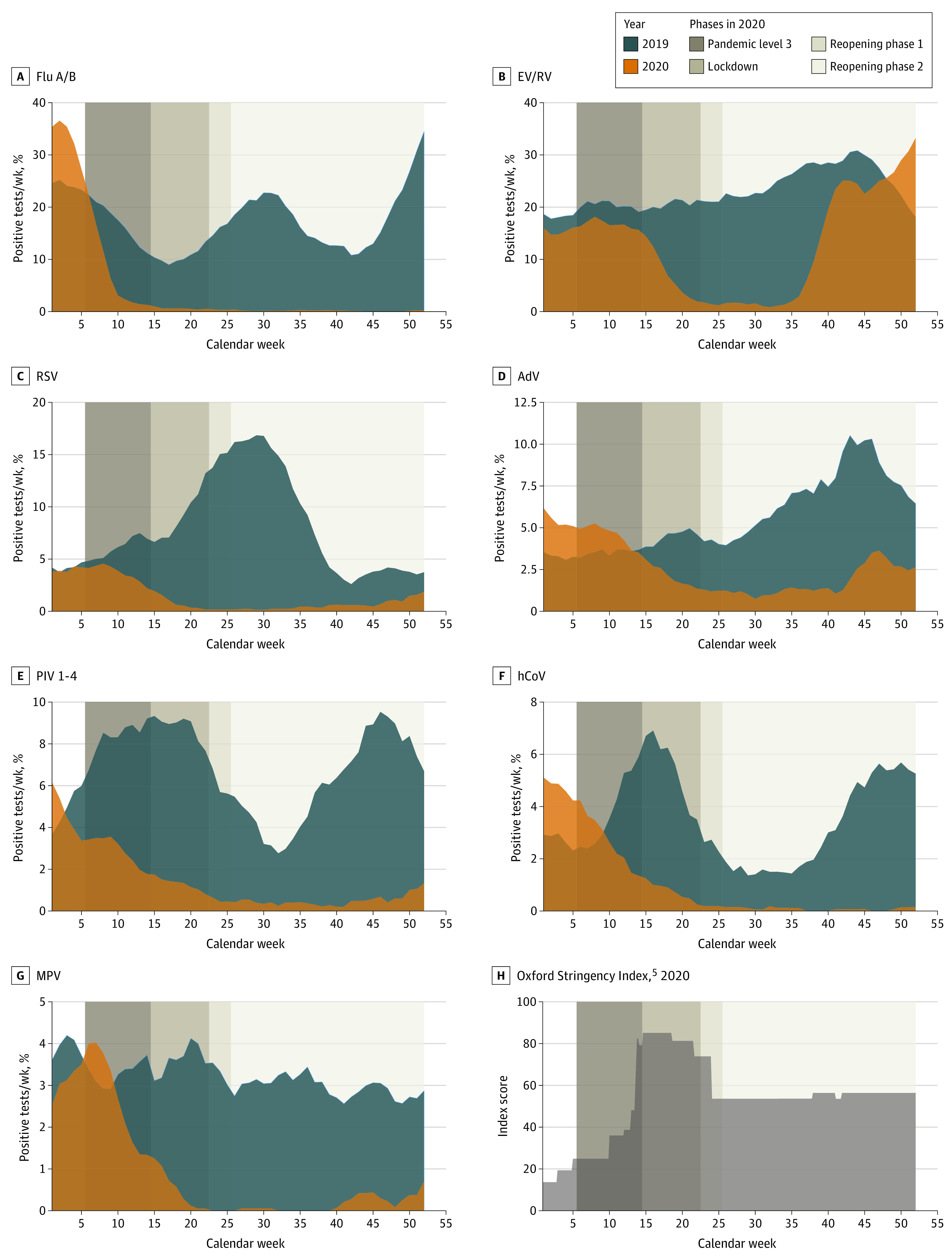
Percentage of Positive Tests for Different Respiratory Viruses in 2019 and in 2020 AdV indicates adenovirus; EV/RV, enterovirus/rhinovirus (combined test); flu A/B, influenza virus A/B; hCoV, common cold coronaviruses HKU1, NL63, 229E, and OC43; MPV, human metapneumovirus; PIV 1-4, parainfluenza viruses 1-4; and RSV, respiratory syncytial virus.

Implementation of prelockdown measures was associated with a reduction of influenza and most other viruses (Figure 1 and Figure 2).^5^ However, a decrease of enterovirus/rhinovirus and adenovirus was only observed during lockdown, when movement restrictions were implemented (eg, prelockdown, enterovirus/rhinovirus, week 1-14, 2020: mean [SD], 16.1 [2.6] cases per 100 samples; lockdown, week 15-22, 2020: mean [SD], 3.9 [3.1] cases per 100 samples; *P* < .001). This phase saw substantial decreases across all respiratory viruses.

During reopening, low levels of all viruses were sustained for approximately 13 weeks, but a reemergence of enterovirus/rhinovirus occurred in early September and a less pronounced rebound of adenovirus in mid-October (enterovirus/rhinovirus, week 15-36, 2020: mean [SD], 2.6 [2.4] cases per 100 samples; week 37-52, 2020: mean 27.1 [7.2] cases per 100 samples; *P* < .001; adenovirus, week 15-42, 2020: mean 1.3 [0.6] cases per 100 samples; week 43-52, 2020: mean 3.1 [1.0] cases per 100 samples; *P* < .001). Most other viruses remained at historically low levels until the end of the year, despite reopening businesses and schools (with mask wearing) and gradual resumption of activities. Small increases for RSV, parainfluenza, and metapneumovirus were seen in November and December.

## Discussion

COVID-19 pandemic control measures in Singapore were associated with changes across a broad range of respiratory viruses; the changes were most substantial during lockdown. However, the patterns of decrease and subsequent reemergence differed between viruses and pandemic response phases.

A decrease in influenza was already seen following relatively modest control measures, including mask-wearing recommendations among only ill or symptomatic individuals. Influenza remained nearly absent for the remainder of 2020. International travel restrictions likely contributed to this.

For enterovirus/rhinovirus and adenovirus, reductions were only seen after lockdown, and these viruses rebounded earlier than others. Continued mandatory mask wearing did not appear to prevent this. These are small, hydrophilic, nonenveloped viruses that are hardier than others, and we hypothesize that greater relative propensity for contact transmission, in addition to droplet transmission, at a time of increasing social contacts during reopening may have contributed to these findings.^6^ The multitude of different enterovirus/rhinovirus and adenovirus types may be another factor contributing to their reemergence.

This study has limitations. The data were obtained from routine diagnostic tests and thus depend on prevailing test requesting patterns. Changes in health care–seeking behavior during the pandemic may have influenced the trends observed.

In conclusion, Singapore’s COVID-19 response represents a unique situation of effectively enforced populationwide interventions that resulted in a broad decrease in the transmission of respiratory viruses. With phased relaxation of measures, different viruses showed different propensities for reemergence, with most enveloped viruses remaining contained at a time of continued universal mask wearing. Further studies into these phenomena are a matter of public health importance; this should include assessing the changes into 2021, differences between adults and children, and associating changes more precisely with different pandemic control measures and their relaxation.

## Notes

Supplement. **eTable.** Timeline of Pandemic Response and Lockdown Measures, Singapore 2020 **eMethods.** Supplementary Methods eReferences.
