# Supplementary material for: Trends in Respiratory Virus Infections During the COVID-19 Pandemic in Singapore, 2020
Source: JAMA Netw Open. 2021 Jun 28;4(6):e2115973. doi: 10.1001/jamanetworkopen.2021.15973 (PMC8239970; doi:10.1001/jamanetworkopen.2021.15973)
Supplement: Supplement. — eTable. Timeline of Pandemic Response and Lockdown Measures, Singapore 2020 eMethods. Supplementary Methods eReferences. [file jamanetwopen-e2115973-s001.pdf]

Supplemental Online Content

Wan WY, Thoon KC, Loo LH, et al. Trends in respiratory virus infections during the COVID-19 pandemic in Singapore, 2020. *JAMA Netw Open.* 2021;4(6):e2115973. doi:10.1001/jamanetworkopen.2021.15973

eTable. Timeline of Pandemic Response and Lockdown Measures, Singapore 2020

eMethods. Supplementary Methods

eReferences.

This supplemental material has been provided by the authors to give readers additional information about their work.

© 2021 Wan WY et al. *JAMA Network Open.*

eTable. Timeline of Pandemic Response and Lockdown Measures, Singapore 2020[a]

Week[b]	Phase	Key events or measures
4	NA	• First imported case reported
6	Pandemic level 3 (DORSCON Orange)	• Non-essential large events (>1,000 people) deferred<br>• Daily health monitoring at workplace (including temperature)<br>• Businesses encouraged to adopt telecommuting<br>• Schools to suspend external activities and large gatherings<br>• Preschools and social services limit number of visitors<br>• Public reminded of personal hygiene and social distancing<br>• Persons who are unwell to seek medical attention and wear masks
13		• Social gatherings limited to 10 persons<br>• Closure of entertainment venues (e.g. cinemas, bars)<br>• International travel restrictions and stay-home notices for returning residents
15	Lockdown ("Circuit Breaker")	• F&B outlets: no dine-in, only takeaways/deliveries<br>• Suspension of non-essential businesses<br>• Restricted commute, work from home encouraged<br>• School closures – move to full home-based learning<br>• Full closure of recreational venues (e.g. museums, theme parks)<br>• Re-usable masks distributed free to all households<br>• Use of masks strongly encouraged outside of home<br>• Deployment of public Safe Distancing Ambassadors
16		• Use of masks mandatory outside of home (anyone >2 years old)
17		• Further restriction on businesses, e.g. hairdressers<br>• Temperature screening & contact traceability logs at all outlets<br>• No social home visits/gatherings
23	Re-opening Phase 1	• Phased re-opening of selected businesses<br>• Phased re-opening of schools
25-52	Re-opening Phase 2	• F&B outlets: dine-in resumed; no more than 5 persons per table<br>• Safe distancing measures remain in place<br>• Masks outside of home remain compulsory

Abbreviations: NA, not applicable; DORSCON, Disease Outbreak Response System Condition; F&B, food and beverage.
[a] Source: Ministry of Health Singapore and Wikipedia.[1,2]
[b] Calendar (epidemiological) week in 2020.

© 2021 Wan WY et al. *JAMA Network Open.*

eMethods. Supplementary Methods

Data from respiratory virus multiplex PCRs performed during 2019 and 2020 were obtained from three public hospitals in Singapore: Hospital A, the only public specialist women's and children's hospital (800 beds), Hospital B, a public hospital offering pediatric and adult medical services (1200 beds), and Hospital C, the largest adult public hospital (1700 beds). These three hospitals represent about 40% of all public hospital beds.[3] Weekly numbers of tests and positive results for each virus were collated; all were de-identified without patient demographics.

Specimens from patients with respiratory symptoms, sent for routine diagnostic purposes, were tested using the FilmArray Respiratory Panels (BioFire, Salt Lake City, UT, USA; Hospital A, entire period; Hospital B, from May 2020), the NxTAG Respiratory Pathogen Panel (Luminex, Austin, TX, USA; Hospital B, until May 2020) and the Anyplex II RV16 Detection kit (Seegene, Seoul, South Korea; Hospital C, entire period).

Institutional Review Board (IRB) application and review was not required under SingHealth institutional rules, because the study constitutes a surveillance/outbreak investigation study that was stripped of all identifying information.

Analysis of weekly numbers of tests and positive results for each virus was performed using Microsoft Excel software (Redmond, WA, USA). Analysis and depiction of the weekly numbers of positive tests for each virus, including 5-week trailing averages, and the Oxford Stringency Index, was performed using the R programming language and software for statistical computing (https://www.r-project.org). For reporting, we followed the STROBE Statement for cross-sectional studies, as applicable to our study.[4]

A prepublication version of this article has been deposited in MedRxiv.[5]

© 2021 Wan WY et al. *JAMA Network Open.*

eReferences

1. Ministry of Health Singapore. Past updates on COVID-19 local situation. https://www.moh.gov.sg/covid-19/past-updates. Accessed November 4, 2020.
2. Wikipedia. Timeline of the COVID-19 pandemic in Singapore. https://en.wikipedia.org/wiki/Timeline_of_the_COVID-19_pandemic_in_Singapore. Accessed January 11, 2021.
3. Ministry of Health Singapore. Beds in inpatient facilities and places in non-residential long-term care facilities. https://www.moh.gov.sg/resources-statistics/singapore-health-facts/beds-in-inpatient-facilities-and-places-in-non-residential-long-term-care-facilities. Accessed November 4, 2020.
4. von Elm E, Altman DG, Egger M, Pocock SJ, Gøtzsche PC, Vandenbroucke JP, STROBE Initiative. The Strengthening the Reporting of Observational Studies in Epidemiology (STROBE) statement: guidelines for reporting observational studies. PLoS Med. 2007 Oct 16;4(10):e296. https://doi.org/10.1371/journal.pmed.0040296.
5. Wan WY, Thoon KC, Loo LH, Chan KS, Oon LLE, Ramasamy A, Maiwald M. Broad decline and subsequent differential re-emergence of respiratory viruses during COVID-19 pandemic response measures, Singapore 2020 (pre-print). MedRxiv. 2021. https://doi.org/10.1101/2021.03.23.21251968.

© 2021 Wan WY et al. *JAMA Network Open.*
